# Supplementary figures and images for: Batch normalization followed by merging is powerful for phenotype prediction integrating multiple heterogeneous studies
Source: PLoS Comput Biol. 2023 Oct 16;19(10):e1010608. doi: 10.1371/journal.pcbi.1010608 (PMC10602384; doi:10.1371/journal.pcbi.1010608)

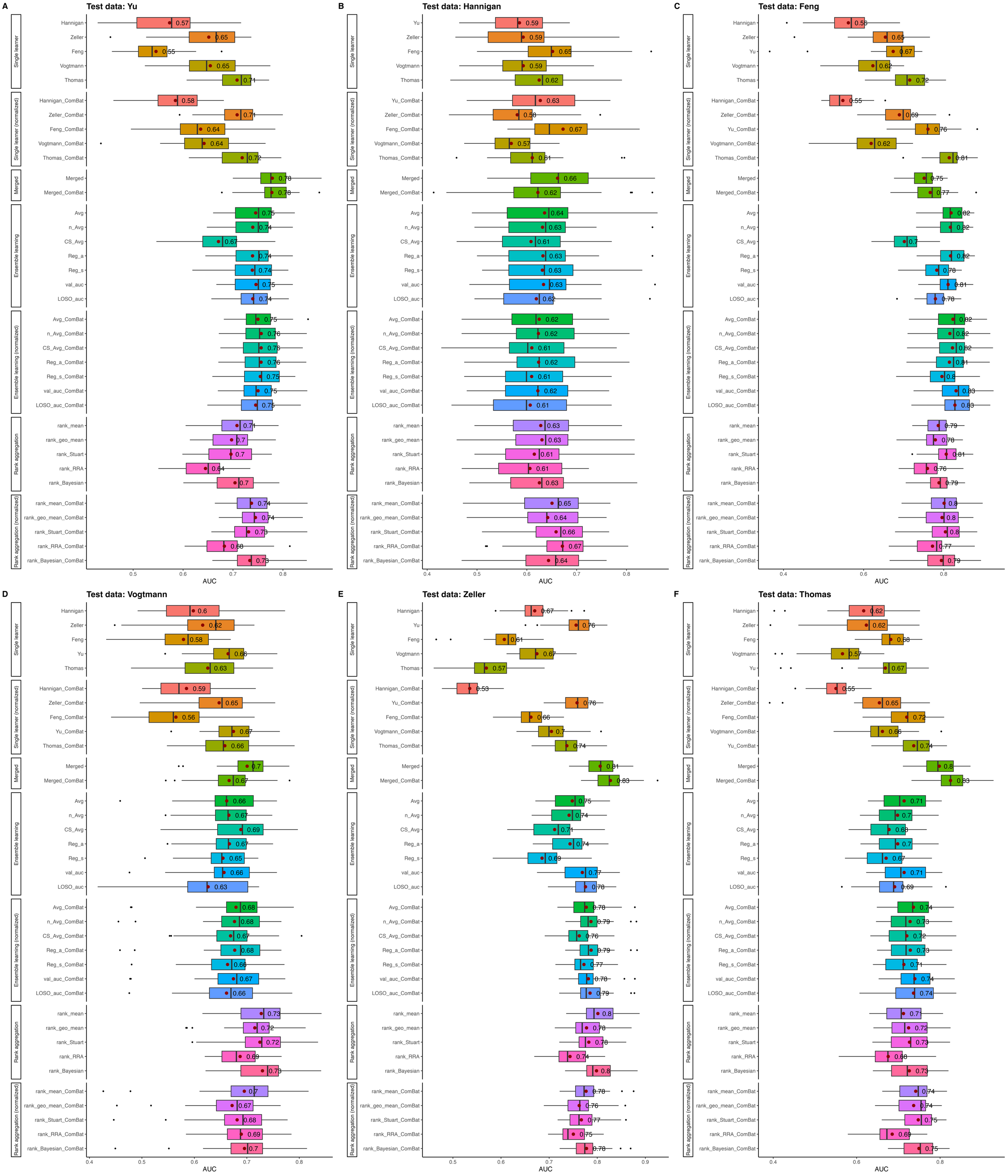

Supplement: S1 Fig — The results by different methods are grouped into six groups. “Single learner”: Each of the five training datasets were trained independently with RF classifier and predicted on the test dataset, then the average AUC score was taken among the five predictions. “Merged”: Merging method with pooling all five training datasets into one training data. The “Single learner” and “Merged” experiments were conducted under both naive and ComBat normalization settings. “Ensemble learning”: The five training predictors were integrated by ensemble weighted learning methods under naive setting. “Ensemble learning (normalized)”: The five training predictors were integrated by ensemble weighted learning methods under ComBat normalization setting. “Rank aggregation”: The five training predictors were integrated by rank aggregation methods under naive setting. “Rank aggregation (normalized)”: The five training predictors were integrated by rank aggregation methods under ComBat normalization setting. The red dots and associated values on the figure are the mean AUC scores for each method, the vertical bars are the median AUC scores for each method, while the black dots represent the outliers. Same method under different settings are represented in the same color of boxplots. All the experiments were repeated 30 times for each test dataset. (TIF) [file pcbi.1010608.s001.tif]

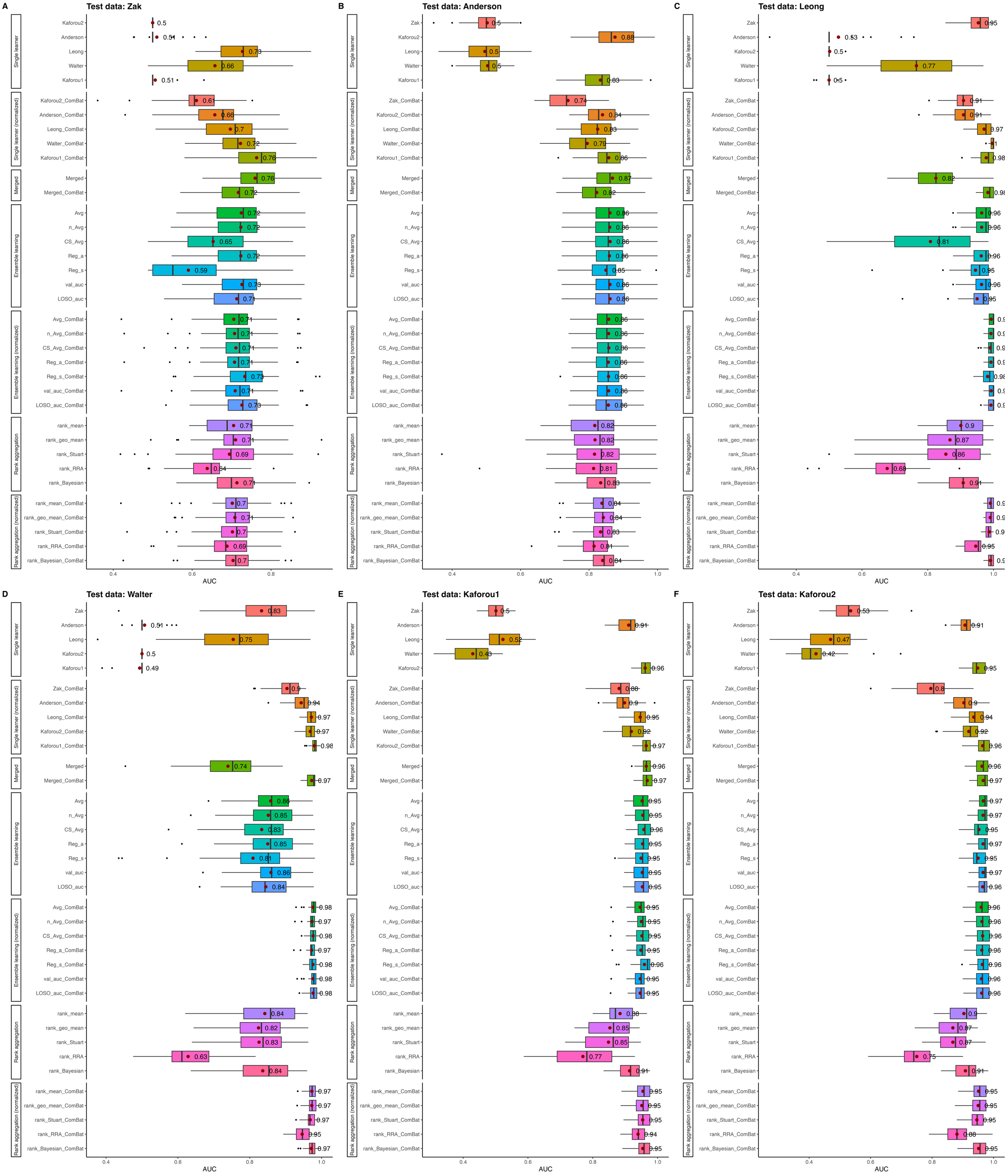

Supplement: S2 Fig — The results by different methods are grouped into six groups. “Single learner”: Each of the five training datasets were trained independently with RF classifier and predicted on the test dataset, then the average AUC score was taken among the five predictions. “Merged”: Merging method with pooling all five training datasets into one training data. The “Single learner” and “Merged” experiments were conducted under both naive and ComBat normalization settings. “Ensemble learning”: The five training predictors were integrated by ensemble weighted learning methods under naive setting. “Ensemble learning (normalized)”: The five training predictors were integrated by ensemble weighted learning methods under ComBat normalization setting. “Rank aggregation”: The five training predictors were integrated by rank aggregation methods under naive setting. “Rank aggregation (normalized)”: The five training predictors were integrated by rank aggregation methods under ComBat normalization setting. The red dots and associated values on the figure are the mean AUC scores for each method, the vertical bars are the median AUC scores for each method, while the black dots represent the outliers. Same method under different settings are represented in the same color of boxplots. All the experiments were repeated 30 times for each test. (TIF) [file pcbi.1010608.s002.tif]

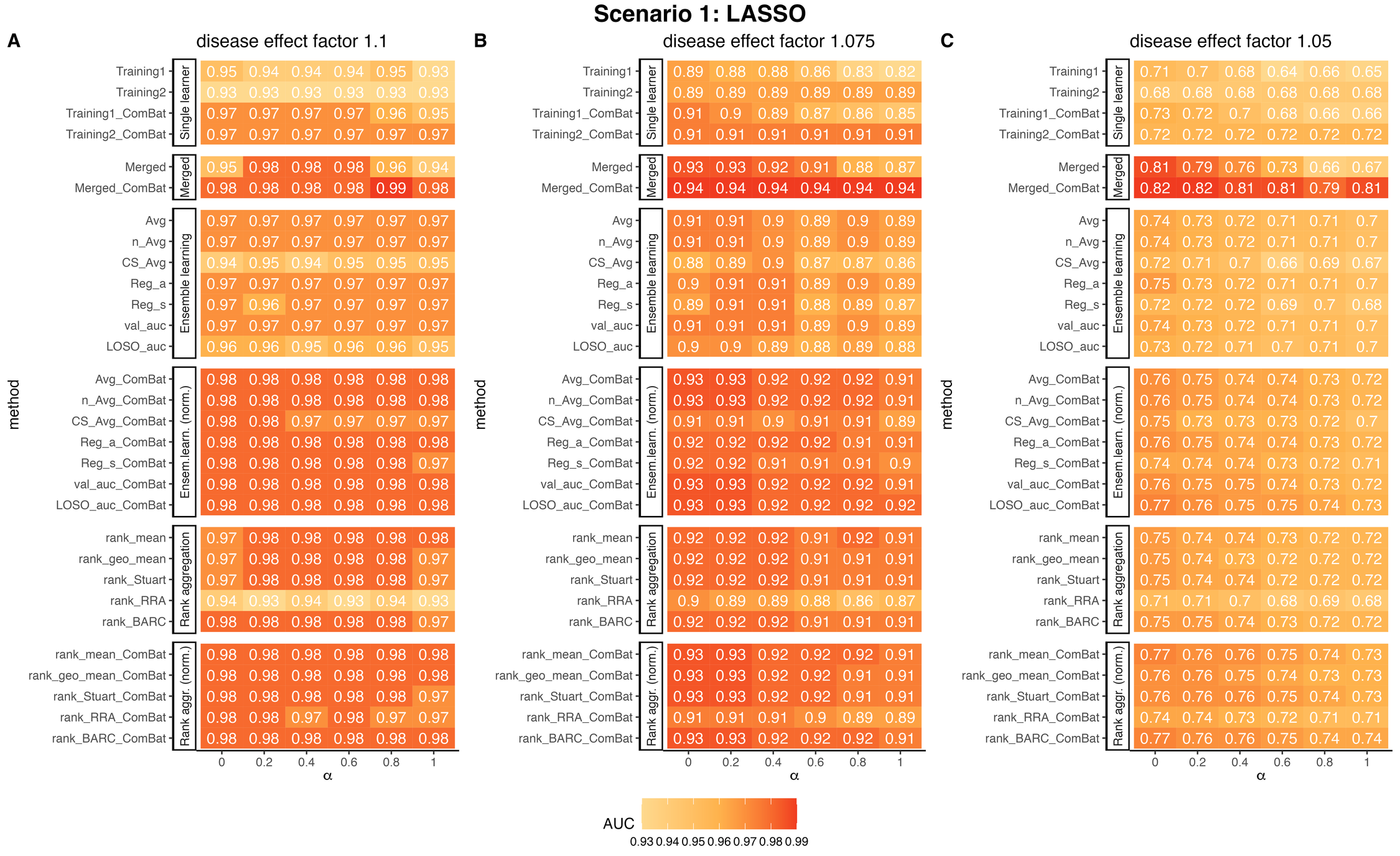

Supplement: S3 Fig — The figures show the AUCs predictions of LASSO using different integration methods with three different disease effect factors. Columns represents different values of α. All the method names without a suffix of “ComBat” are the methods carried out in the naive setting, while the names with a suffix of “ComBat” were carried out in the ComBat normalization setting. All the experiments were repeated for 100 times and the AUC scores shown on the figure are the averages from the 100 trials. However, the differences between the results using normalization versus no-normalization are not as dramatic as other machine learning classifiers indicating robustness of LASSO with respect to population differences. (TIF) [file pcbi.1010608.s003.tif]

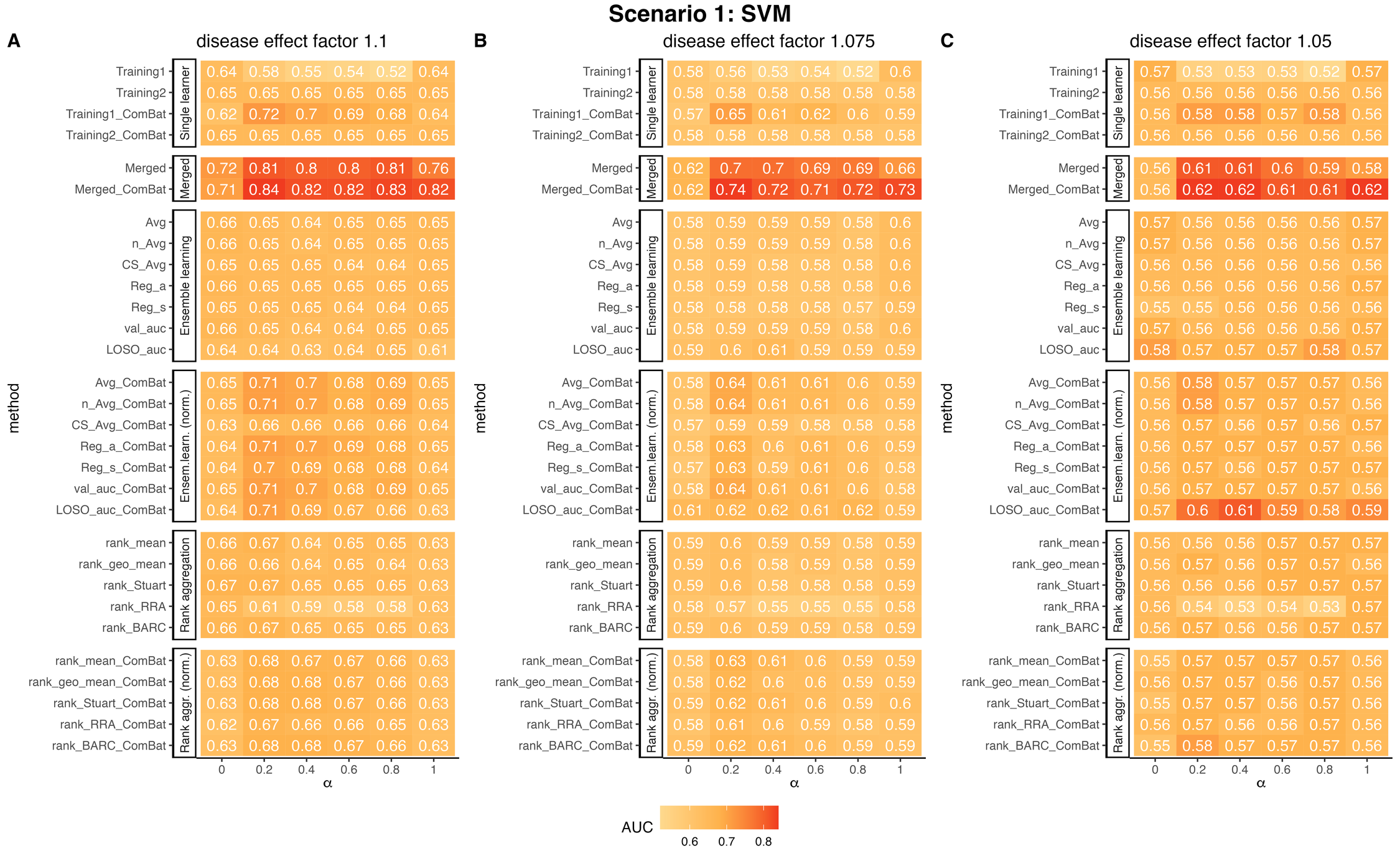

Supplement: S4 Fig — The figures show the AUCs predictions of SVM with polynomial kernel using different integration methods with three different disease effect factors. Columns represents different values of α. All the method names without a suffix of “ComBat” are the methods carried out in the naive setting, while the names with a suffix of “ComBat” were carried out in the ComBat normalization setting. All the experiments were repeated for 100 times and the AUC scores shown on the figure are the averages from the 100 trials. (TIF) [file pcbi.1010608.s004.tif]

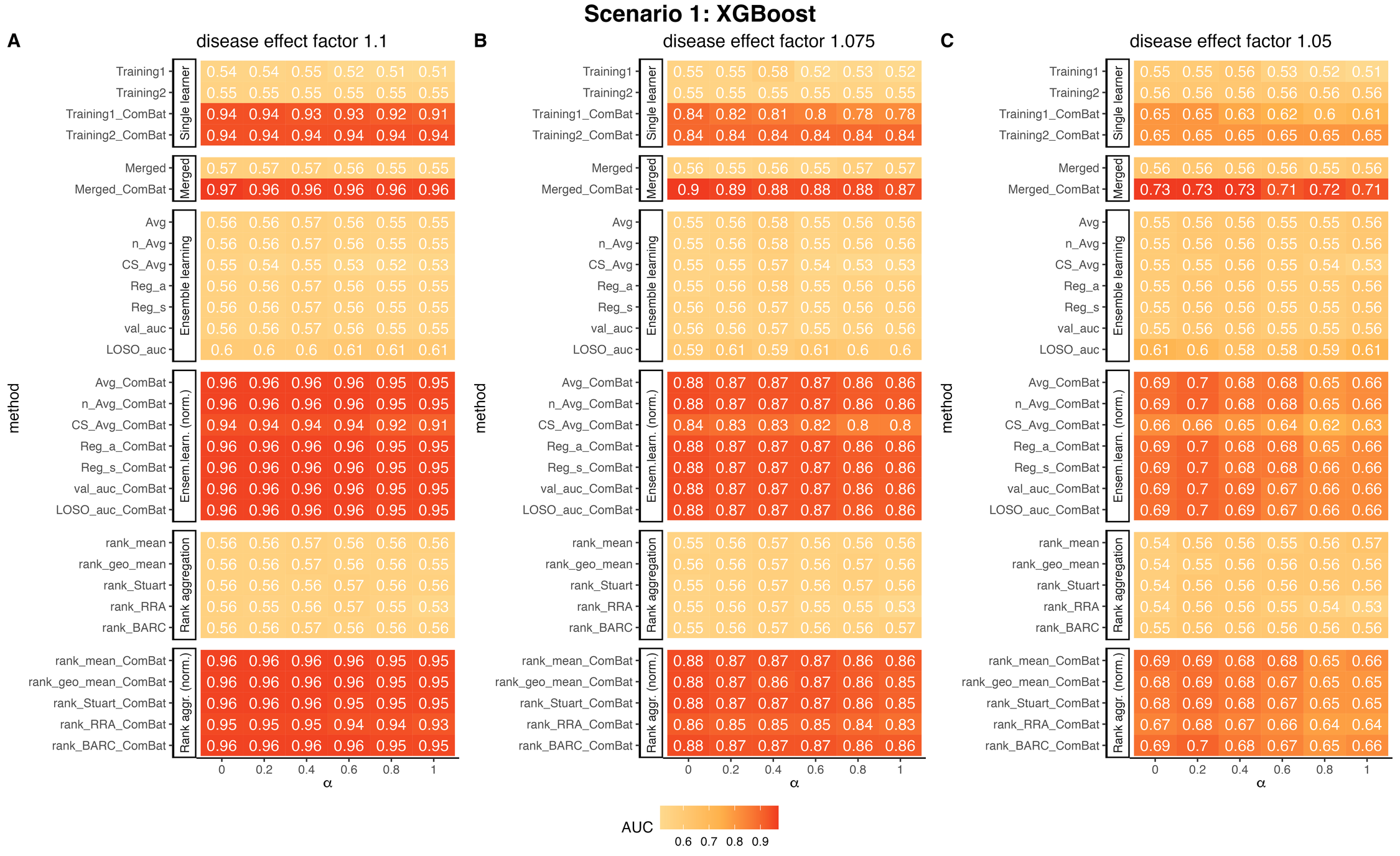

Supplement: S5 Fig — The figures show the AUCs predictions of XGBoost using different integration methods with three different disease effect factors. Columns represents different values of α. All the method names without a suffix of “ComBat” are the methods carried out in the naive setting, while the names with a suffix of “ComBat” were carried out in the ComBat normalization setting. All the experiments were repeated for 100 times and the AUC scores shown on the figure are the averages from the 100 trials. (TIF) [file pcbi.1010608.s005.tif]

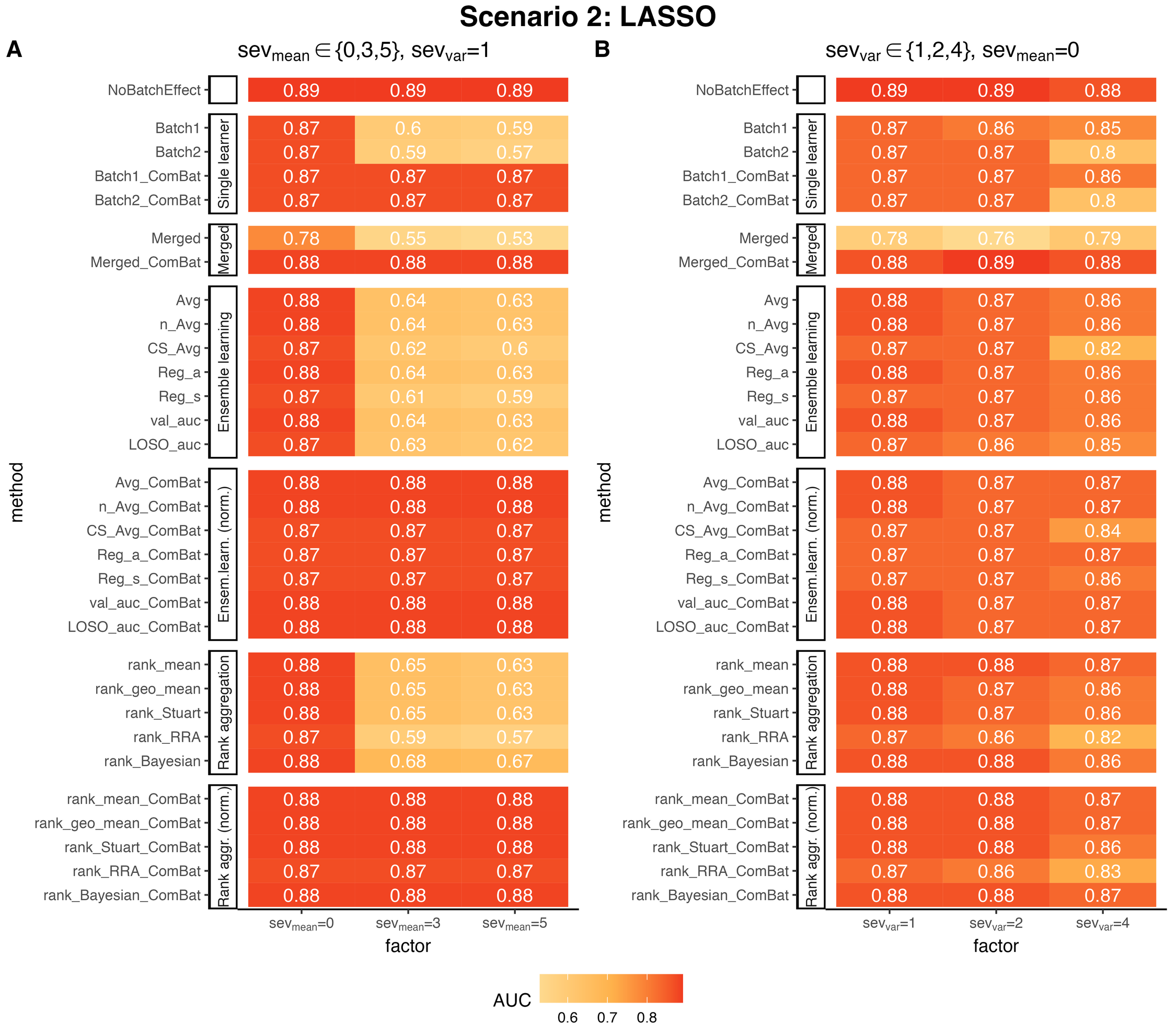

Supplement: S6 Fig — The figures show the AUCs predictions of LASSO using different integration methods with various batch severity levels. A: AUC score comparisons with different severity levels of additive batch effects on the mean of OTU abundances, with no multiplicative batch effect on the variance. B: AUC score comparisons with different severity levels of multiplicative batch effects on the variance of OTU abundances, with no additive batch effect on the mean. The disease effect factor was set to 1.025 for both situations. All the method names without a suffix of “ComBat” are the methods done in naive setting, while the names with a suffix of “ComBat” were done in ComBat normalization setting. All the experiments were repeated for 100 times and the AUC scores shown on the figure are the averages from the 100 trials. (TIF) [file pcbi.1010608.s006.tif]

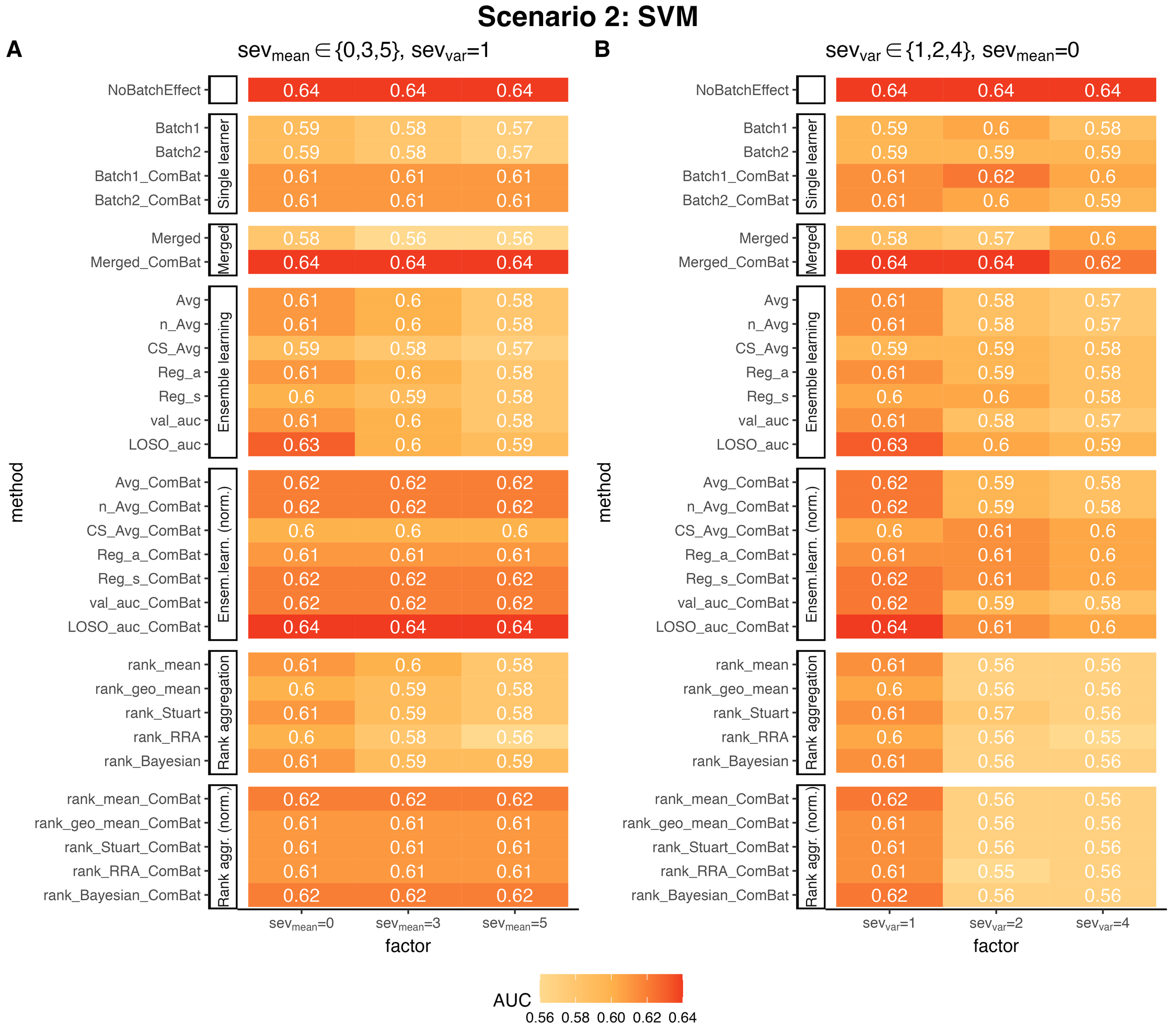

Supplement: S7 Fig — The figures show the AUCs predictions of SVM with polynomial kernel using different integration methods with various batch severity levels. A: AUC score comparisons with different severity levels of additive batch effects on the mean of OTU abundances, with no multiplicative batch effect on the variance. B: AUC score comparisons with different severity levels of multiplicative batch effects on the variance of OTU abundances, with no additive batch effect on the mean. The disease effect factor was set to 1.025 for both situations. All the method names without a suffix of “ComBat” are the methods done in naive setting, while the names with a suffix of “ComBat” were done in ComBat normalization setting. All the experiments were repeated for 100 times and the AUC scores shown on the figure are the averages from the 100 trials. (TIF) [file pcbi.1010608.s007.tif]

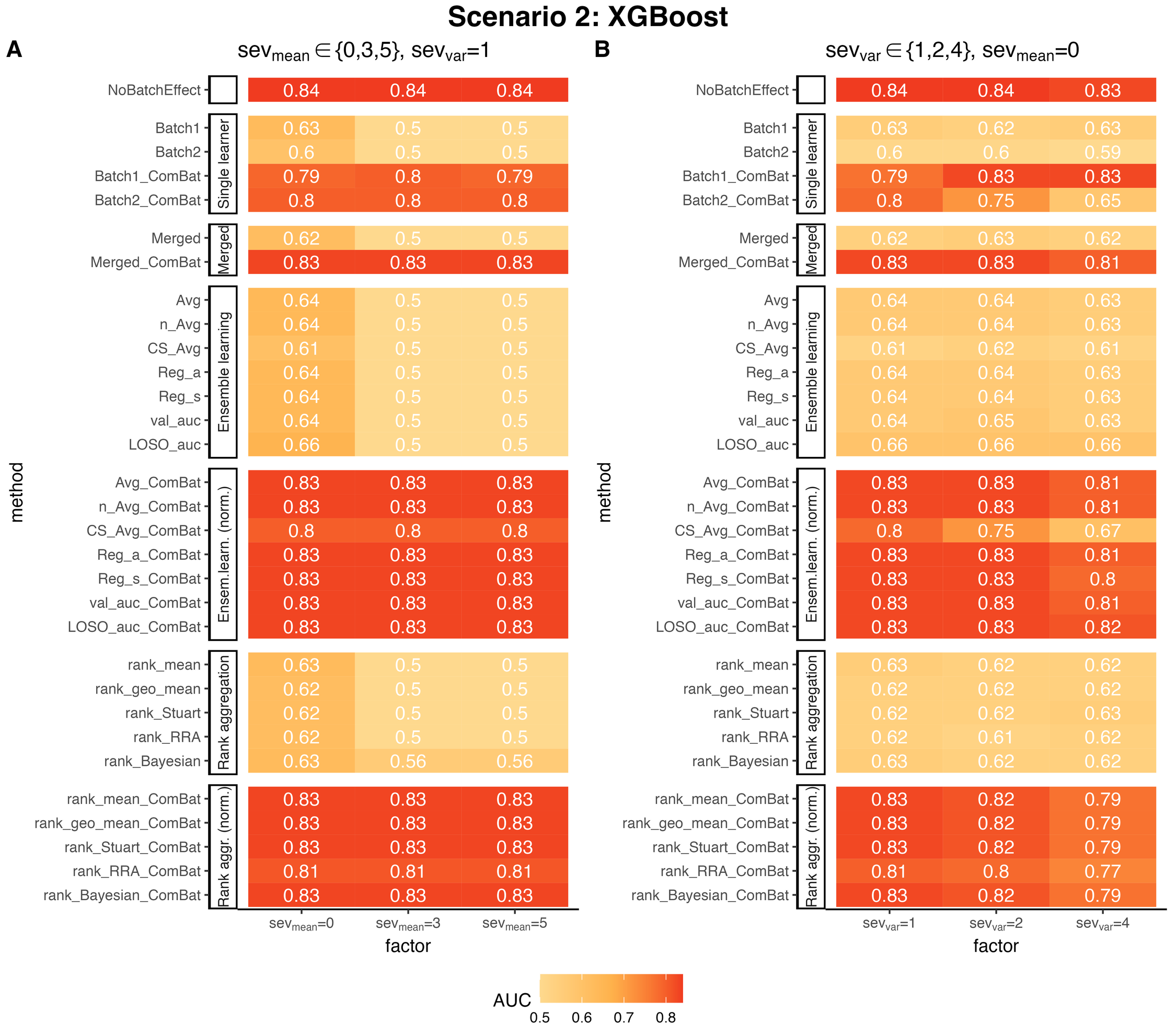

Supplement: S8 Fig — The figures show the AUCs predictions of XGBoost using different integration methods with various batch severity levels. A: AUC score comparisons with different severity levels of additive batch effects on the mean of OTU abundances, with no multiplicative batch effect on the variance. B: AUC score comparisons with different severity levels of multiplicative batch effects on the variance of OTU abundances, with no additive batch effect on the mean. The disease effect factor was set to 1.025 for both situations. All the method names without a suffix of “ComBat” are the methods done in naive setting, while the names with a suffix of “ComBat” were done in ComBat normalization setting. All the experiments were repeated for 100 times and the AUC scores shown on the figure are the averages from the 100 trials. (TIF) [file pcbi.1010608.s008.tif]

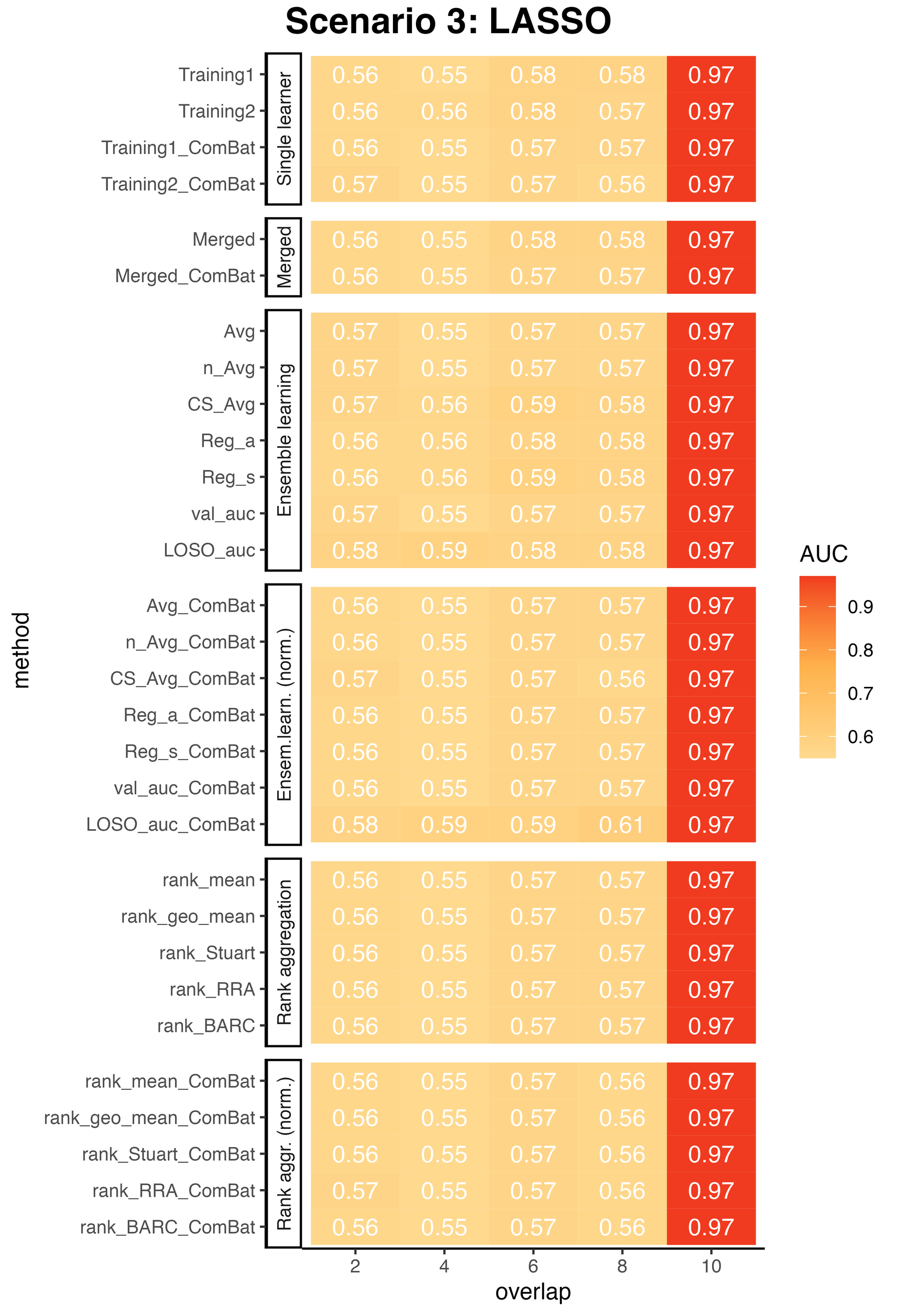

Supplement: S9 Fig — The figures show the AUCs predictions of LASSO using different integration methods with various number of overlapping disease associated OTUs. The disease effect factor was set to 1.075. Columns represent different numbers of overlapping disease associated OTUs in the training and test data, the larger the number, the more similar the two disease models are. When the number achieves 10, the two models are the same in the training and test data. All the experiments were repeated for 100 times and the AUC scores shown on the figure are the averages from the 100 trials. (TIF) [file pcbi.1010608.s009.tif]

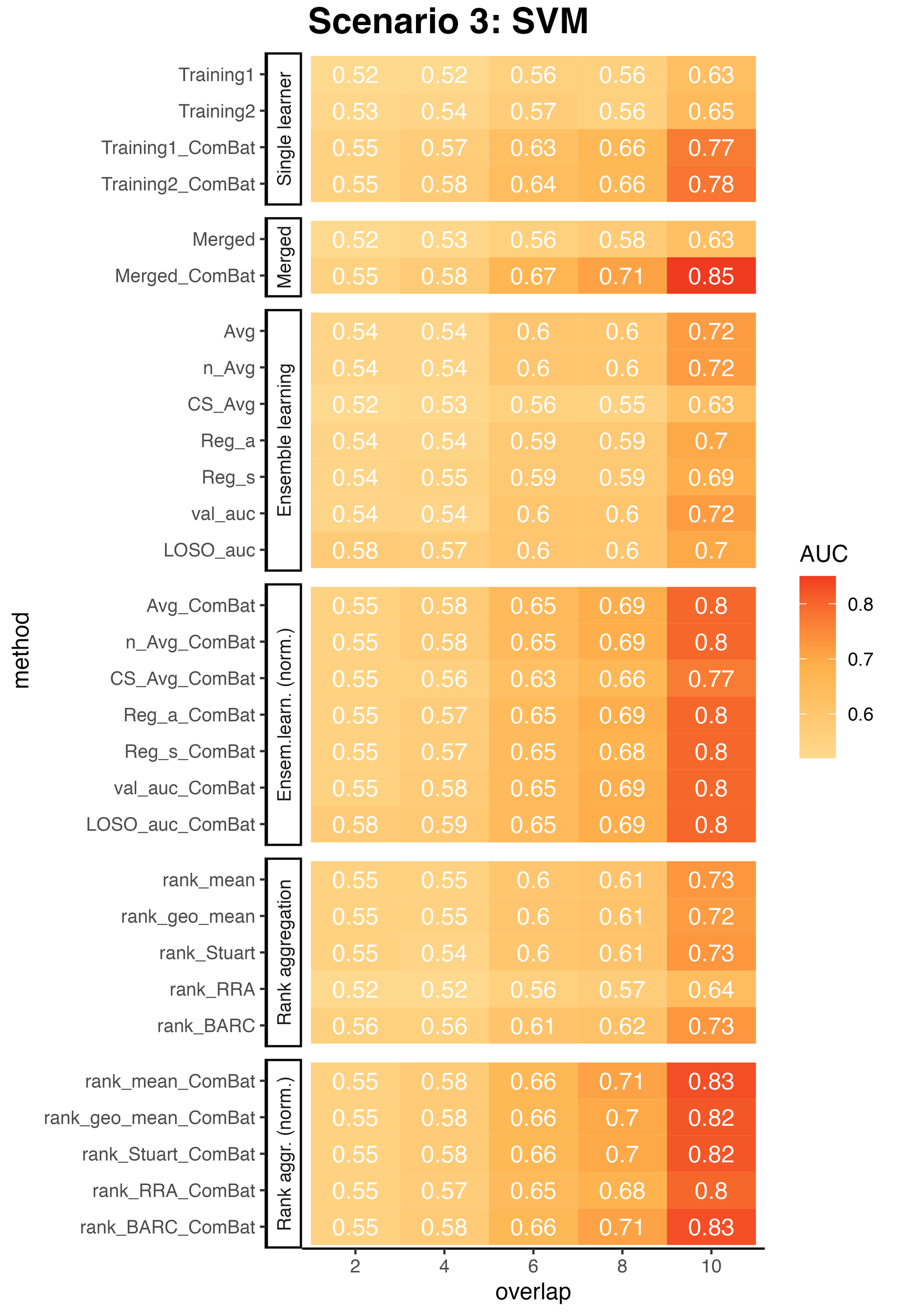

Supplement: S10 Fig — The figures show the AUCs predictions of SVM with polynomial kernel using different integration methods with various number of overlapping disease associated OTUs. The disease effect factor was set to 1.075. Columns represent different numbers of overlapping disease associated OTUs in the training and test data, the larger the number, the more similar the two disease models are. When the number achieves 10, the two models are the same in the training and test data. All the experiments were repeated for 100 times and the AUC scores shown on the figure are the averages from the 100 trials. (TIF) [file pcbi.1010608.s010.tif]

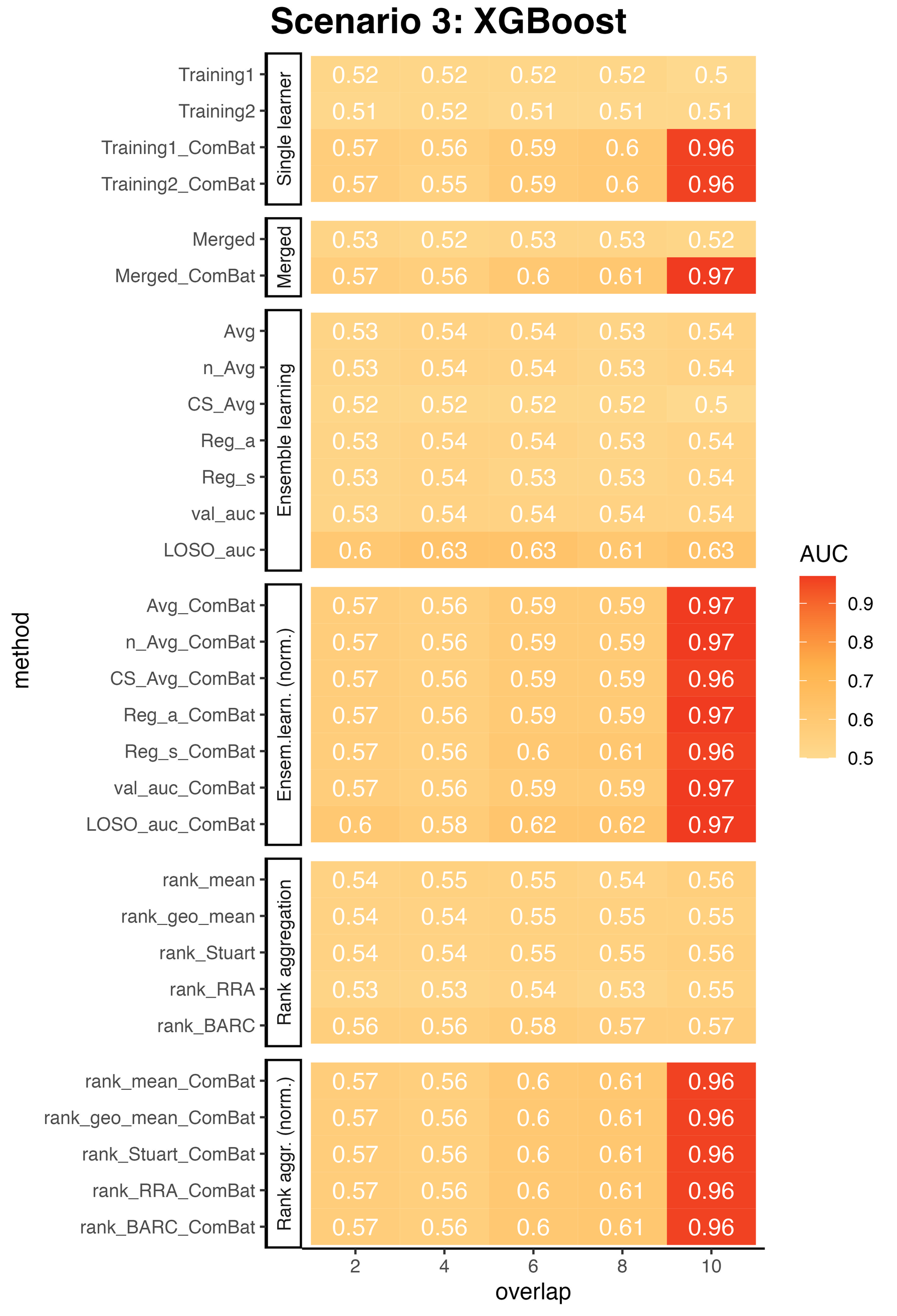

Supplement: S11 Fig — The figures show the AUCs predictions of XGBoost using different integration methods with various number of overlapping disease associated OTUs. The disease effect factor was set to 1.075. Columns represent different numbers of overlapping disease associated OTUs in the training and test data, the larger the number, the more similar the two disease models are. When the number achieves 10, the two models are the same in the training and test data. All the experiments were repeated for 100 times and the AUC scores shown on the figure are the averages from the 100 trials. (TIF) [file pcbi.1010608.s011.tif]

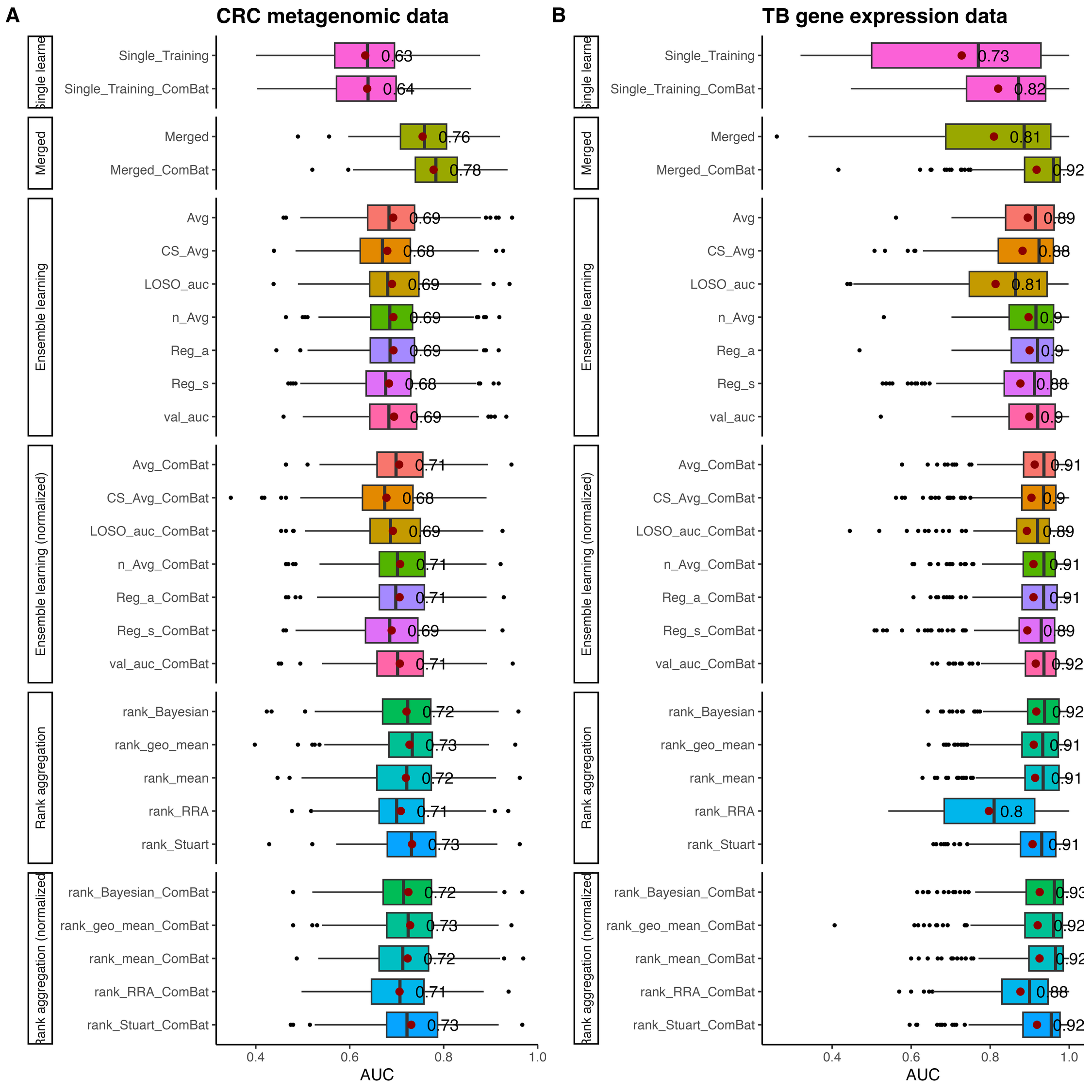

Supplement: S12 Fig — A: Leave-one-dataset-out average AUC score comparisons among different methods in colorectal cancer metagenomic datasets. B: Leave-one-dataset-out average AUC score comparisons among different methods in tuberculosis gene expression datasets. The results by different methods are grouped into six groups. “Single learner”: Each of the five training datasets were trained independently with RF classifier and predicted on the test dataset, then the average AUC score was taken among the five predictions. “Merged”: Merging method with pooling all five training datasets into one training data. The “Single learner” and “Merged” experiments were conducted under both naive and ComBat normalization settings. “Ensemble learning”: The five training predictors were integrated by ensemble weighted learning methods under naive setting. “Ensemble learning (normalized)”: The five training predictors were integrated by ensemble weighted learning methods under ComBat normalization setting. “Rank aggregation”: The five training predictors were integrated by rank aggregation methods under naive setting. “Rank aggregation (normalized)”: The five training predictors were integrated by rank aggregation methods under ComBat normalization setting. The red dots and associated values on the figure are the mean AUC scores for each method, the vertical bars are the median AUC scores for each method, while the black dots represent the outliers. Same method under different settings are represented in the same color of boxplots. All the experiments were repeated 30 times for each test dataset, and the results presented in the figure were based on the average AUC scores of the total 180 replications for the six test datasets for metagenomic and gene expression studies, respectively. (TIF) [file pcbi.1010608.s012.tif]

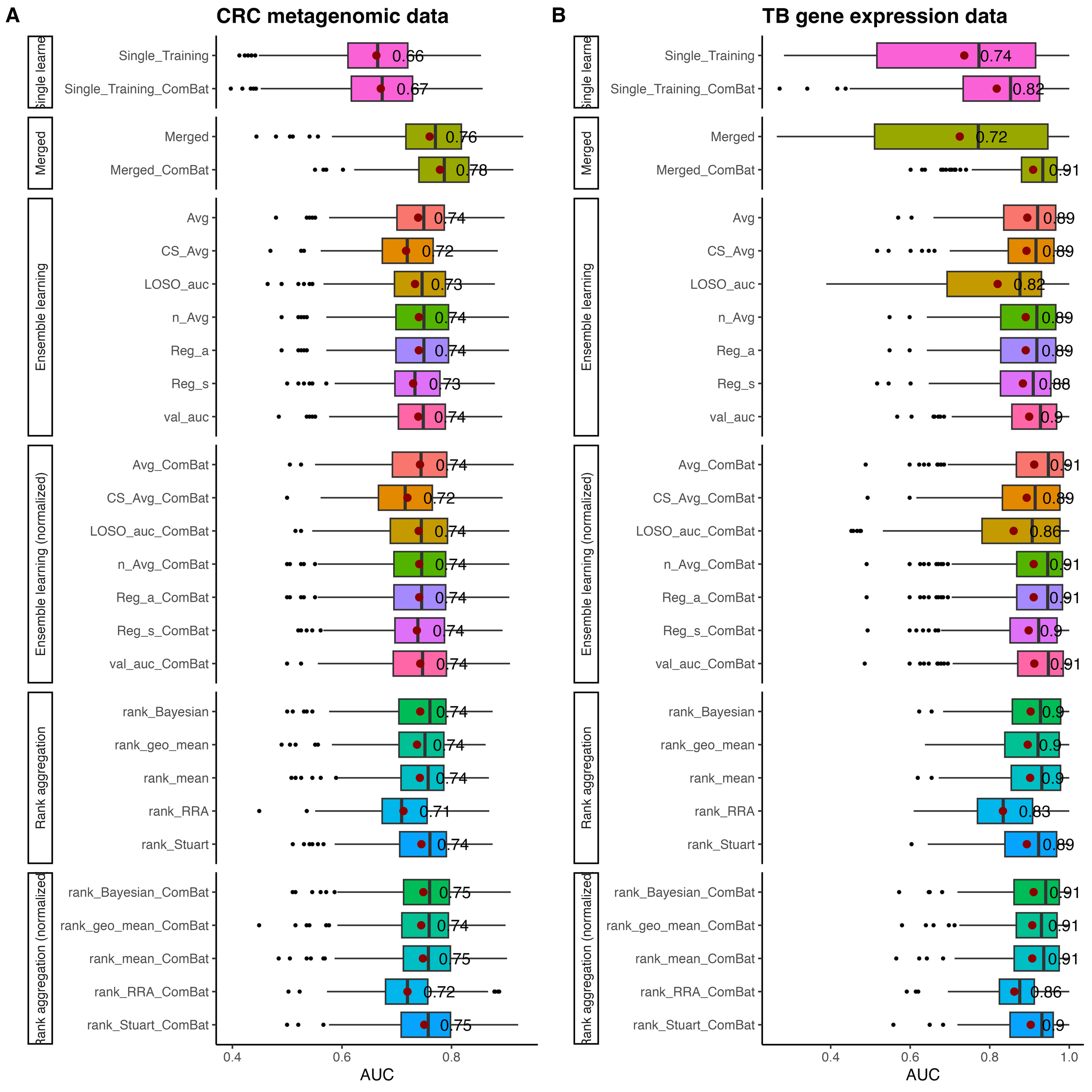

Supplement: S13 Fig — A: Leave-one-dataset-out average AUC score comparisons among different methods in colorectal cancer metagenomic datasets. B: Leave-one-dataset-out average AUC score comparisons among different methods in tuberculosis gene expression datasets. The results by different methods are grouped into six groups. “Single learner”: Each of the five training datasets were trained independently with RF classifier and predicted on the test dataset, then the average AUC score was taken among the five predictions. “Merged”: Merging method with pooling all five training datasets into one training data. The “Single learner” and “Merged” experiments were conducted under both naive and ComBat normalization settings. “Ensemble learning”: The five training predictors were integrated by ensemble weighted learning methods under naive setting. “Ensemble learning (normalized)”: The five training predictors were integrated by ensemble weighted learning methods under ComBat normalization setting. “Rank aggregation”: The five training predictors were integrated by rank aggregation methods under naive setting. “Rank aggregation (normalized)”: The five training predictors were integrated by rank aggregation methods under ComBat normalization setting. The red dots and associated values on the figure are the mean AUC scores for each method, the vertical bars are the median AUC scores for each method, while the black dots represent the outliers. Same method under different settings are represented in the same color of boxplots. All the experiments were repeated 30 times for each test dataset, and the results presented in the figure were based on the average AUC scores of the total 180 replications for the six test datasets for metagenomic and gene expression studies, respectively. (TIF) [file pcbi.1010608.s013.tif]

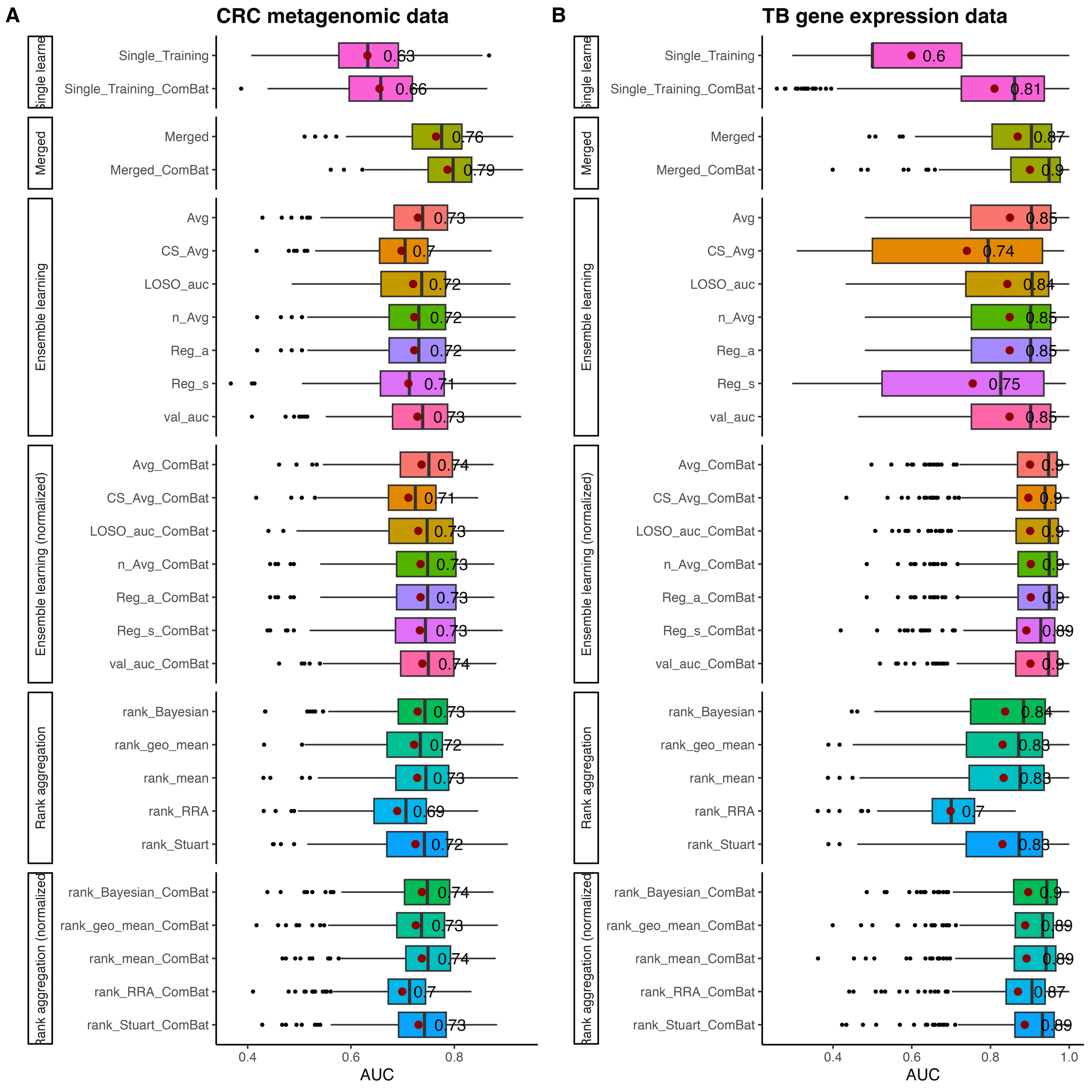

Supplement: S14 Fig — A: Leave-one-dataset-out average AUC score comparisons among different methods in colorectal cancer metagenomic datasets. B: Leave-one-dataset-out average AUC score comparisons among different methods in tuberculosis gene expression datasets. The results by different methods are grouped into six groups. “Single learner”: Each of the five training datasets were trained independently with RF classifier and predicted on the test dataset, then the average AUC score was taken among the five predictions. “Merged”: Merging method with pooling all five training datasets into one training data. The “Single learner” and “Merged” experiments were conducted under both naive and ComBat normalization settings. “Ensemble learning”: The five training predictors were integrated by ensemble weighted learning methods under naive setting. “Ensemble learning (normalized)”: The five training predictors were integrated by ensemble weighted learning methods under ComBat normalization setting. “Rank aggregation”: The five training predictors were integrated by rank aggregation methods under naive setting. “Rank aggregation (normalized)”: The five training predictors were integrated by rank aggregation methods under ComBat normalization setting. The red dots and associated values on the figure are the mean AUC scores for each method, the vertical bars are the median AUC scores for each method, while the black dots represent the outliers. Same method under different settings are represented in the same color of boxplots. All the experiments were repeated 30 times for each test dataset, and the results presented in the figure were based on the average AUC scores of the total 180 replications for the six test datasets for metagenomic and gene expression studies, respectively. (TIF) [file pcbi.1010608.s014.tif]

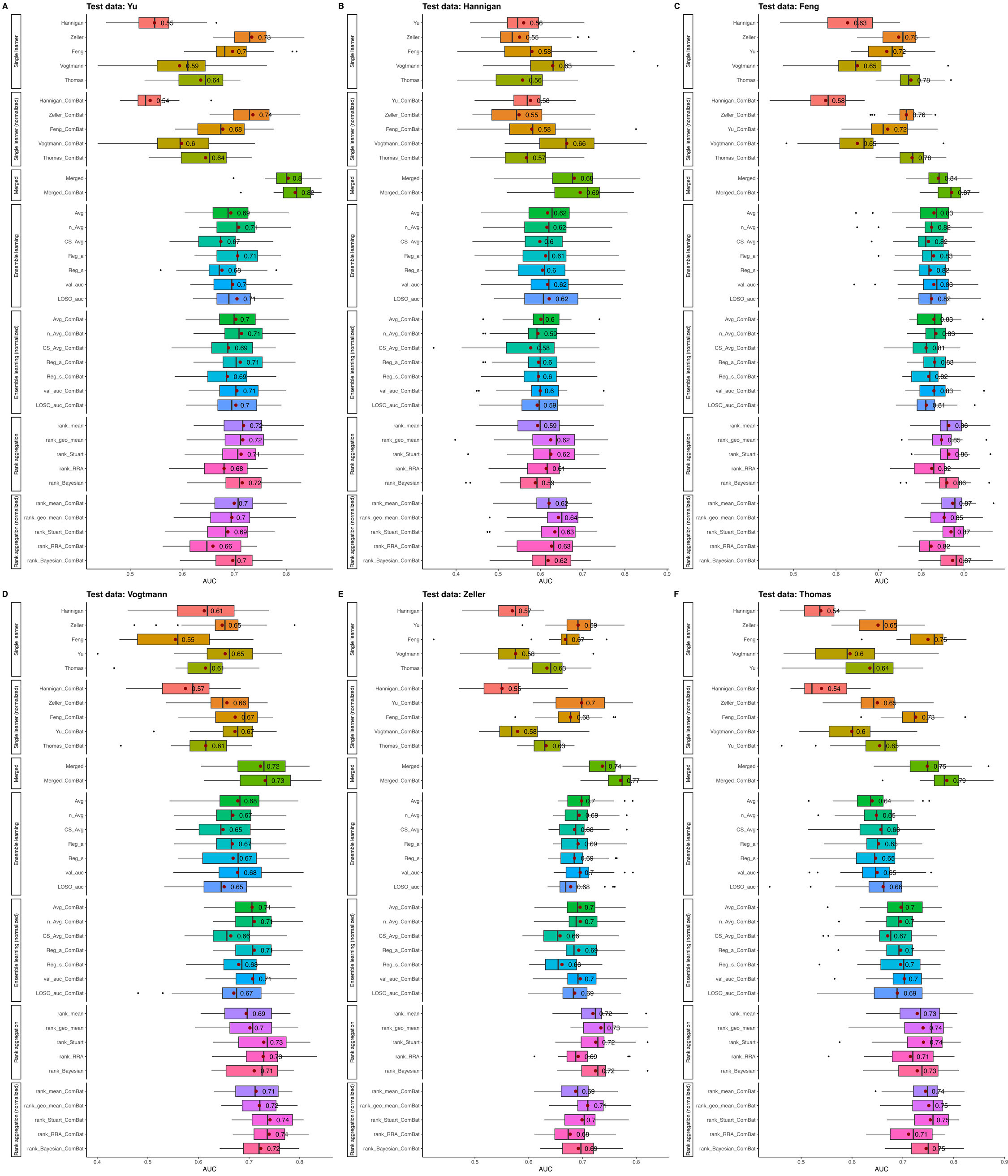

Supplement: S15 Fig — The results by different methods are grouped into six groups. “Single learner”: Each of the five training datasets were trained independently with RF classifier and predicted on the test dataset, then the average AUC score was taken among the five predictions. “Merged”: Merging method with pooling all five training datasets into one training data. The “Single learner” and “Merged” experiments were conducted under both naive and ComBat normalization settings. “Ensemble learning”: The five training predictors were integrated by ensemble weighted learning methods under naive setting. “Ensemble learning (normalized)”: The five training predictors were integrated by ensemble weighted learning methods under ComBat normalization setting. “Rank aggregation”: The five training predictors were integrated by rank aggregation methods under naive setting. “Rank aggregation (normalized)”: The five training predictors were integrated by rank aggregation methods under ComBat normalization setting. The red dots and associated values on the figure are the mean AUC scores for each method, the vertical bars are the median AUC scores for each method, while the black dots represent the outliers. Same method under different settings are represented in the same color of boxplots. All the experiments were repeated 30 times for each test dataset. (TIF) [file pcbi.1010608.s015.tif]

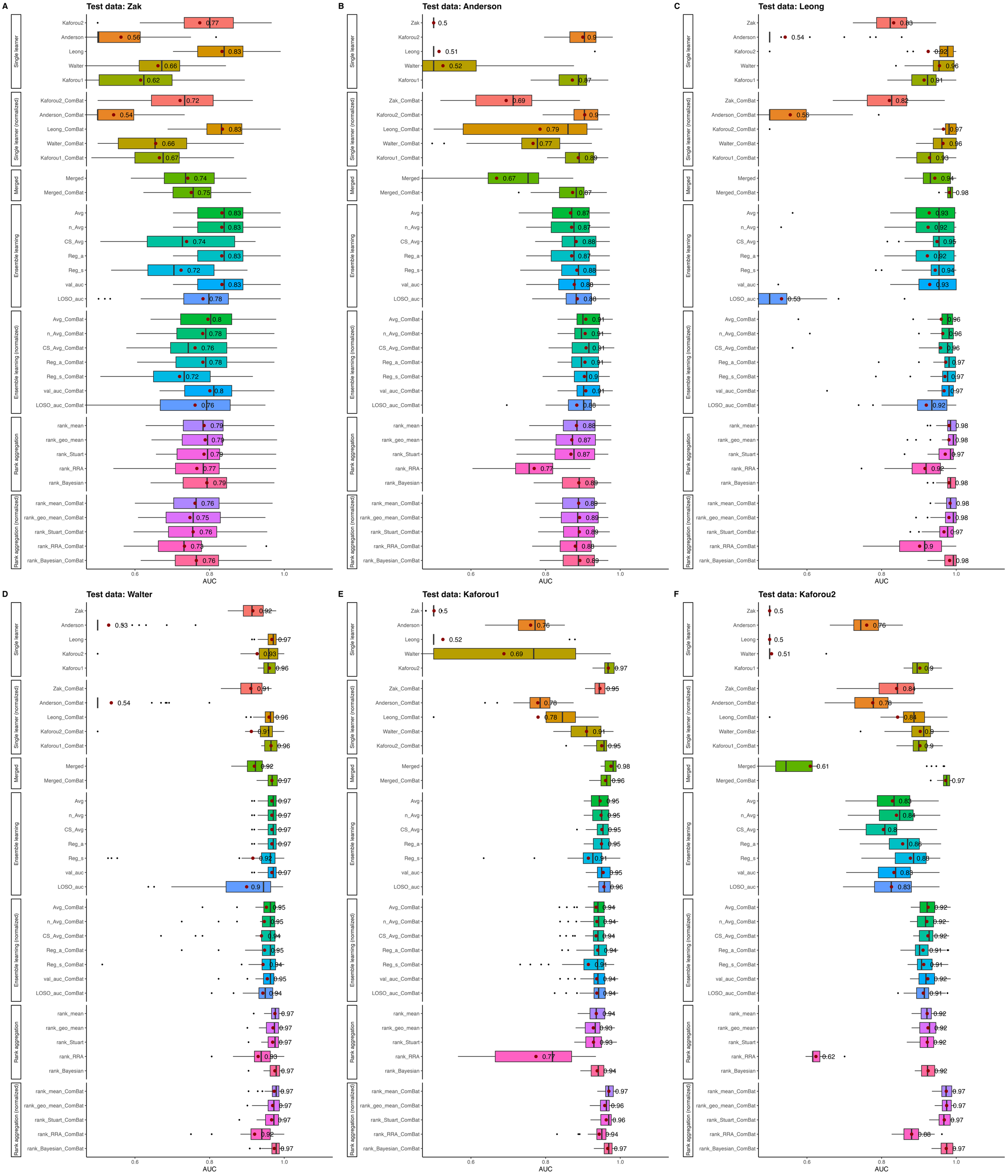

Supplement: S16 Fig — The results by different methods are grouped into six groups. “Single learner”: Each of the five training datasets were trained independently with RF classifier and predicted on the test dataset, then the average AUC score was taken among the five predictions. “Merged”: Merging method with pooling all five training datasets into one training data. The “Single learner” and “Merged” experiments were conducted under both naive and ComBat normalization settings. “Ensemble learning”: The five training predictors were integrated by ensemble weighted learning methods under naive setting. “Ensemble learning (normalized)”: The five training predictors were integrated by ensemble weighted learning methods under ComBat normalization setting. “Rank aggregation”: The five training predictors were integrated by rank aggregation methods under naive setting. “Rank aggregation (normalized)”: The five training predictors were integrated by rank aggregation methods under ComBat normalization setting. The red dots and associated values on the figure are the mean AUC scores for each method, the vertical bars are the median AUC scores for each method, while the black dots represent the outliers. Same method under different settings are represented in the same color of boxplots. All the experiments were repeated 30 times for each test. (TIF) [file pcbi.1010608.s016.tif]

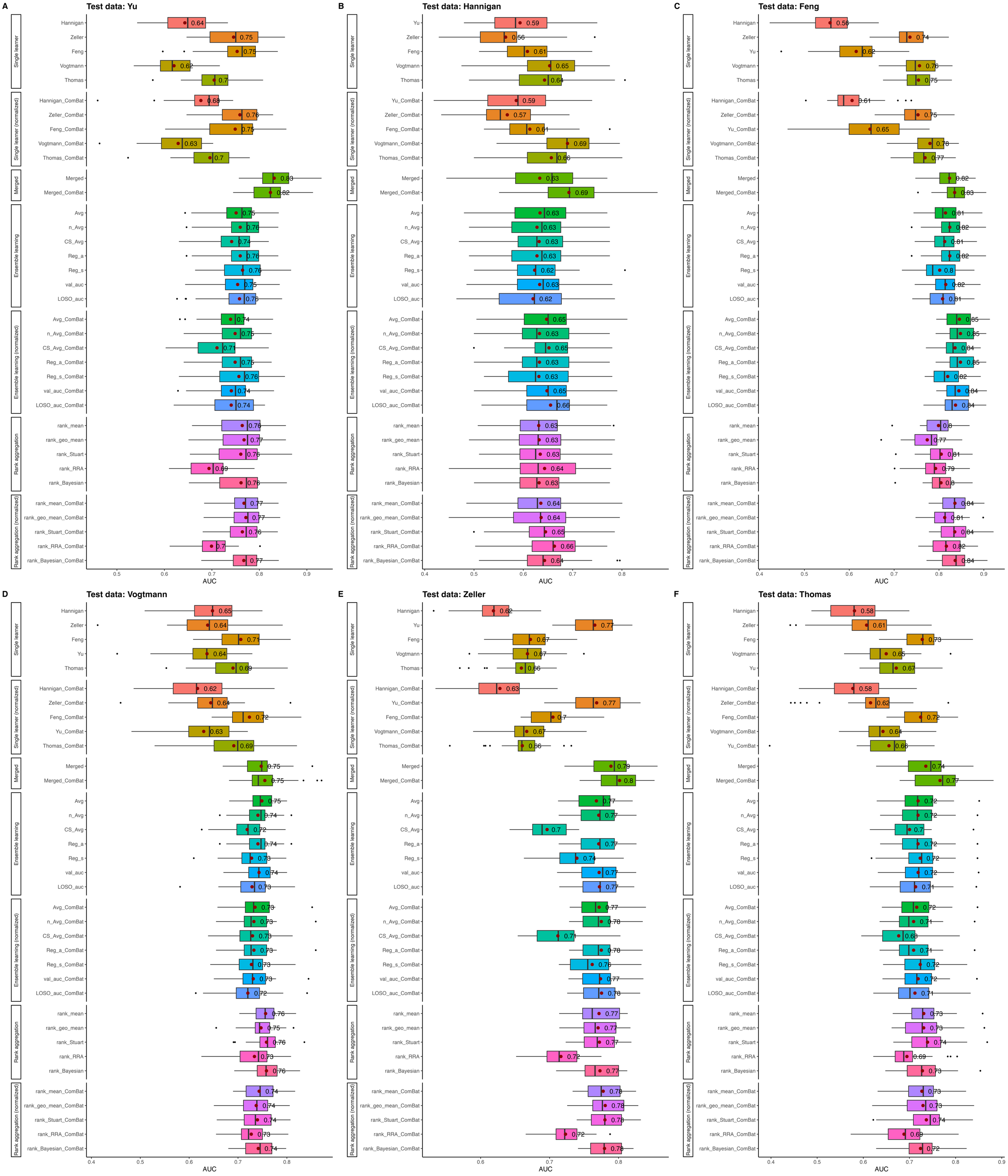

Supplement: S17 Fig — The results by different methods are grouped into six groups. “Single learner”: Each of the five training datasets were trained independently with RF classifier and predicted on the test dataset, then the average AUC score was taken among the five predictions. “Merged”: Merging method with pooling all five training datasets into one training data. The “Single learner” and “Merged” experiments were conducted under both naive and ComBat normalization settings. “Ensemble learning”: The five training predictors were integrated by ensemble weighted learning methods under naive setting. “Ensemble learning (normalized)”: The five training predictors were integrated by ensemble weighted learning methods under ComBat normalization setting. “Rank aggregation”: The five training predictors were integrated by rank aggregation methods under naive setting. “Rank aggregation (normalized)”: The five training predictors were integrated by rank aggregation methods under ComBat normalization setting. The red dots and associated values on the figure are the mean AUC scores for each method, the vertical bars are the median AUC scores for each method, while the black dots represent the outliers. Same method under different settings are represented in the same color of boxplots. All the experiments were repeated 30 times for each test dataset. (TIF) [file pcbi.1010608.s017.tif]

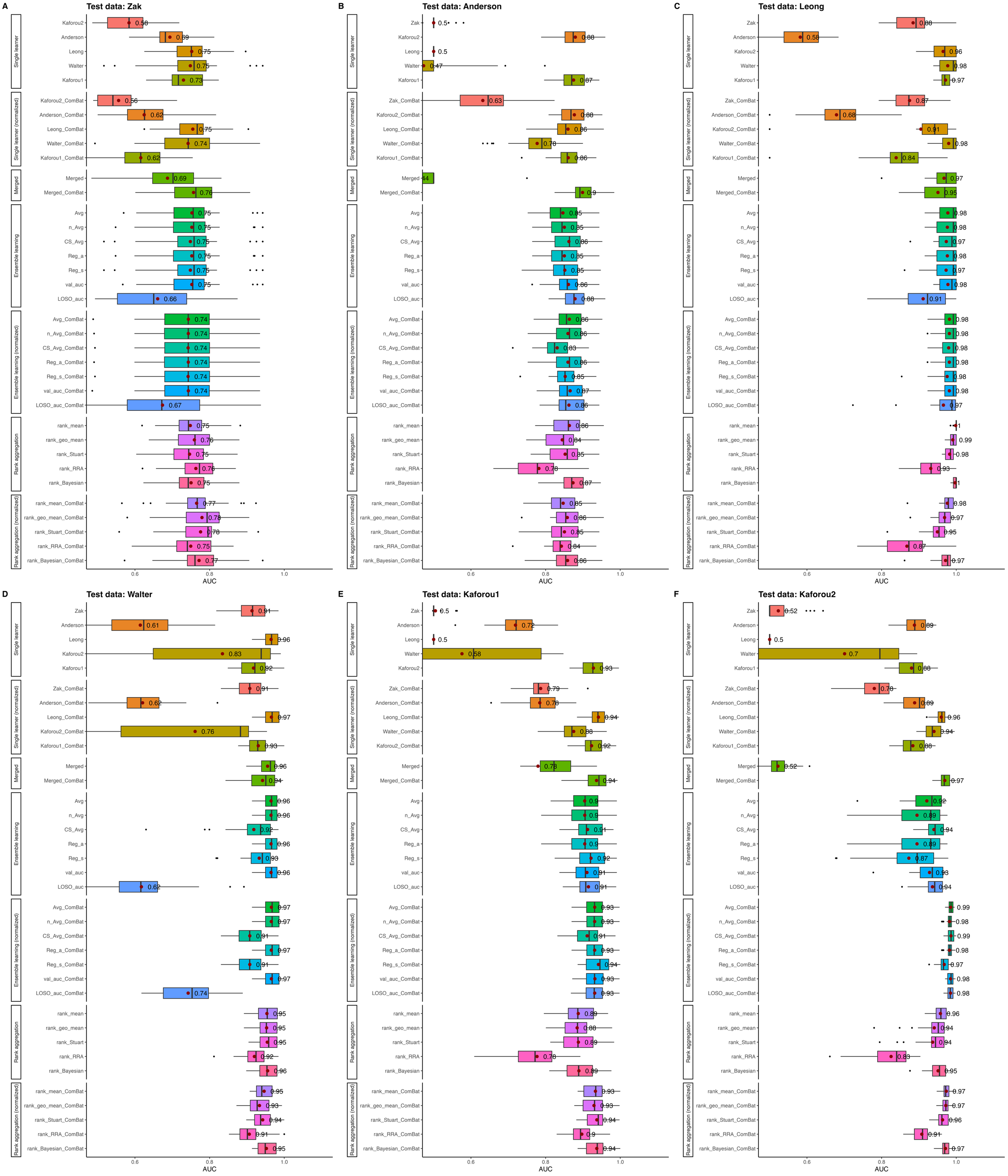

Supplement: S18 Fig — The results by different methods are grouped into six groups. “Single learner”: Each of the five training datasets were trained independently with RF classifier and predicted on the test dataset, then the average AUC score was taken among the five predictions. “Merged”: Merging method with pooling all five training datasets into one training data. The “Single learner” and “Merged” experiments were conducted under both naive and ComBat normalization settings. “Ensemble learning”: The five training predictors were integrated by ensemble weighted learning methods under naive setting. “Ensemble learning (normalized)”: The five training predictors were integrated by ensemble weighted learning methods under ComBat normalization setting. “Rank aggregation”: The five training predictors were integrated by rank aggregation methods under naive setting. “Rank aggregation (normalized)”: The five training predictors were integrated by rank aggregation methods under ComBat normalization setting. The red dots and associated values on the figure are the mean AUC scores for each method, the vertical bars are the median AUC scores for each method, while the black dots represent the outliers. Same method under different settings are represented in the same color of boxplots. All the experiments were repeated 30 times for each test. (TIF) [file pcbi.1010608.s018.tif]

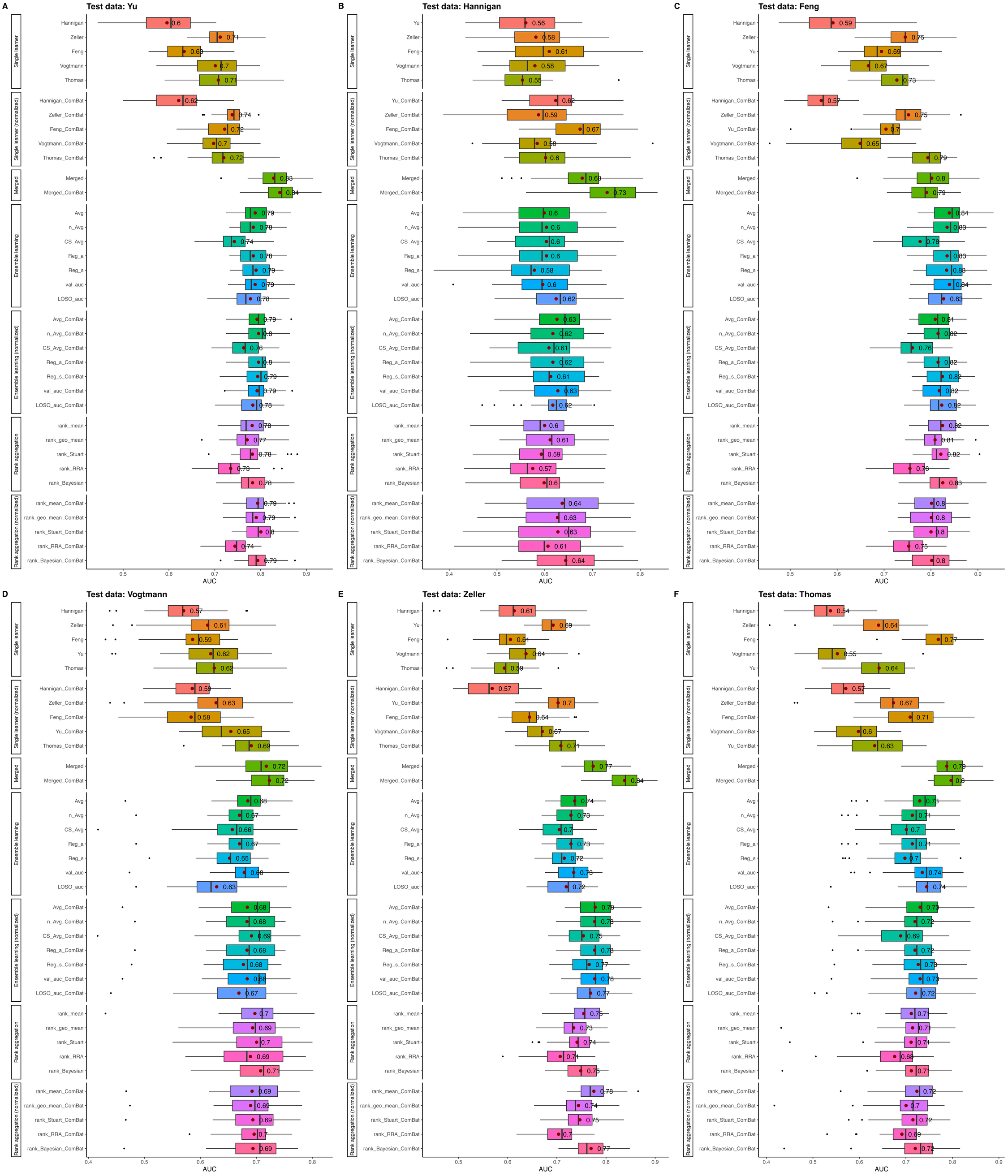

Supplement: S19 Fig — The results by different methods are grouped into six groups. “Single learner”: Each of the five training datasets were trained independently with RF classifier and predicted on the test dataset, then the average AUC score was taken among the five predictions. “Merged”: Merging method with pooling all five training datasets into one training data. The “Single learner” and “Merged” experiments were conducted under both naive and ComBat normalization settings. “Ensemble learning”: The five training predictors were integrated by ensemble weighted learning methods under naive setting. “Ensemble learning (normalized)”: The five training predictors were integrated by ensemble weighted learning methods under ComBat normalization setting. “Rank aggregation”: The five training predictors were integrated by rank aggregation methods under naive setting. “Rank aggregation (normalized)”: The five training predictors were integrated by rank aggregation methods under ComBat normalization setting. The red dots and associated values on the figure are the mean AUC scores for each method, the vertical bars are the median AUC scores for each method, while the black dots represent the outliers. Same method under different settings are represented in the same color of boxplots. All the experiments were repeated 30 times for each test. (TIF) [file pcbi.1010608.s019.tif]

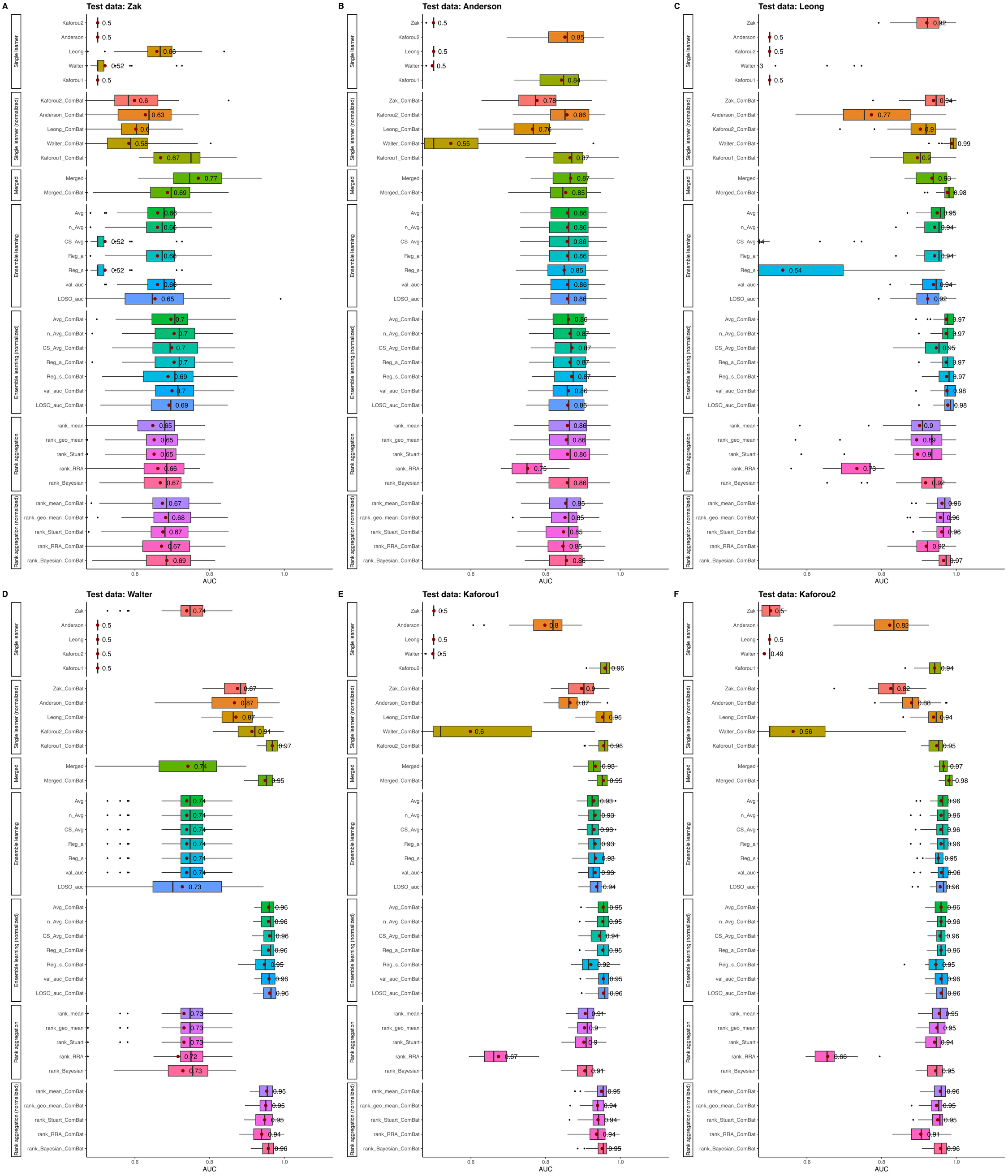

Supplement: S20 Fig — The results by different methods are grouped into six groups. “Single learner”: Each of the five training datasets were trained independently with RF classifier and predicted on the test dataset, then the average AUC score was taken among the five predictions. “Merged”: Merging method with pooling all five training datasets into one training data. The “Single learner” and “Merged” experiments were conducted under both naive and ComBat normalization settings. “Ensemble learning”: The five training predictors were integrated by ensemble weighted learning methods under naive setting. “Ensemble learning (normalized)”: The five training predictors were integrated by ensemble weighted learning methods under ComBat normalization setting. “Rank aggregation”: The five training predictors were integrated by rank aggregation methods under naive setting. “Rank aggregation (normalized)”: The five training predictors were integrated by rank aggregation methods under ComBat normalization setting. The red dots and associated values on the figure are the mean AUC scores for each method, the vertical bars are the median AUC scores for each method, while the black dots represent the outliers. Same method under different settings are represented in the same color of boxplots. All the experiments were repeated 30 times for each test. (TIF) [file pcbi.1010608.s020.tif]
