# Supplementary material for: Batch normalization followed by merging is powerful for phenotype prediction integrating multiple heterogeneous studies
Source: PLoS Comput Biol. 2023 Oct 16;19(10):e1010608. doi: 10.1371/journal.pcbi.1010608 (PMC10602384; doi:10.1371/journal.pcbi.1010608)
Supplement: Attachment — Submitted filename: Response to reviewers.doc [file pcbi.1010608.s021.doc]

Dear Drs. Coelho and Wang,

We greatly appreciate the opportunity for a revision of our manuscript. We also thank all reviewers for their important comments. These suggestions considerably helped us to improve the manuscript. We hope this revision meets the high publication standard of *PLoS Computational Biology*.

In the revision, we have revised our manuscript by carefully considering the reviewers' suggestions and comments and carried out additional experiments to address the major concerns.

In the following section, we will present point-by-point responses to the comments.

Thank you very much for your further consideration of our manuscript.

Sincerely Yours,

Yilin Gao and Fengzhu Sun

Fengzhu Sun, PhD

Professor of Quantitative and Computational Biology

University of Southern California

Los Angeles, CA 90089

[fsun@usc.edu](mailto:fsun@usc.edu)

**Response to reviewer #1**

Thank you very much for your thoughtful and constructive comments! Your comments helped to greatly improve the manuscript. In the revision, we followed all your suggestions and addressed all the points with details shown below (your comments are in bold).

**1. The authors only test one classifier in their study. I wonder whether the same observations applied to some other classifier, e.g. SVM, lasso and XGBoost etc.**

**Response:** Thank you for your valuable comment regarding the use of different classifiers in our study. We agree that examining the results using various classifiers, such as SVM, Lasso, and XGBoost, can indeed enhance the robustness of our findings.

Following your suggestion, we repeated our experiments using these classifiers. We included the methods of these classifiers in the Methods section and added a subsection of “Consistency across LASSO, SVM, and XGBoost classifiers mirroring RF Classifier in simulation studies and real data applications” in the Results section (page 17, line 691), and discussed the results in the Discussion section (page 20, line 857) as well. We found that all three methods displayed similar trends to the original classifier random forests we previously investigated. The consistency across different classification methods strengthens our confidence in the observations reported in our original study.

We sincerely appreciate your insights and believe that this addition further fortifies the validity of our findings.

**2. The gap of the prediction accuracy rates between different approaches are much more dramatic in the simulated data compared to the real data. I am wondering how realistic the simulated datasets are. The authors discuss the potential reason is that as the number of training datasets increase, the gap between different approach will decrease, it will be good if the authors can explore this aspect in the simulation.**

**Response:** Thank you for your insightful query regarding the disparity in prediction accuracy between our simulated and real data. We agree with your observation that the gap in the tested methods is more pronounced in our simulated data than the real data and understand your curiosity about the realism of our simulated datasets.

Our simulation parameters were designed to amplify the impacts of disease effect factors and heterogeneity to vividly demonstrate the advantages of integrating normalization and integration methods. This intentional exaggeration is the main cause for the larger gaps observed in the simulation results than in the real data.

In the real-world applications, we observed less pronounced improvements in prediction accuracy. This was anticipated, and as we discussed in discussion section of the paper (page 19, line 821), it could be attributed to the less pronounced disease effect factor in real-world scenarios and the relatively smaller differences between the training and testing populations compared to our simulations.

In regard to the potential reason, we previously mentioned about the decreasing gap with an increase in the number of training datasets, upon careful evaluation, we have come to realize that this might not be the underlying reason explaining the decrease in gap between different approaches. Therefore, we have removed this aspect from the discussion. Instead, we have included a discussion about the potential reason that disease effects on genomic features between cases and controls may be less pronounced in real-world scenarios, as mentioned above.

We genuinely appreciate your suggestion, as it prompted us to reconsider the potential reasons for the differences between simulations and real studies. Your input has helped us correct our previous statement and provide a clearer explanation of our findings.

**3. While batch normalization can remove the batch effect and study specific variation, the current and improve the phenotype prediction. The current strategies are limited as they require the test data information and thus the training data needs to be re-adjusted and the model needs to be retrained every time when a new test dataset comes in. It will be good that the author can discuss the limitation and also this approach compared to strategies like domain adaption and methods like CPOP (Wang et al. 2022).

• Wang, K.Y.X., Pupo, G.M., Tembe, V. et al. Cross-Platform Omics Prediction procedure: a statistical machine learning framework for wider implementation of precision medicine.npj Digit. Med. 5, 85 (2022).** [**https://doi.org/10.1038/s41746-022-00618-5**](https://urldefense.com/v3/__https:/doi.org/10.1038/s41746-022-00618-5__;!!LIr3w8kk_Xxm!r6t8Pt14XBLiJ8HrIPztF79LcD8LJ_HWWgFbVZaCDmlRjdMY905tX-afDsMLXYg_x-9aE18_SJU5HcxRzaE$) **Response:** We appreciate your thoughtful comment highlighting the limitations of current strategies in our study, particularly the dependency on test data information when using test data abundance as a reference to normalize training data abundance. We also thank you for suggesting the comparison with domain adaptation strategies and methods like CPOP (Wang et al., 2022).

We have added a discussion in our manuscript addressing these points in the Discussion section (page 20, line 863). We acknowledge the limitation of our approach, especially the requirement of re-adjusting training data and retraining the model with the introduction of each new test dataset. We have compared this with the CPOP strategy, which is designed to make predictions on a single sample without the necessity for normalization or additional data integration.

While highlighting these comparative limitations, we have also emphasized the strength of our methodology, particularly its applicability to all the features and flexibility to be integrated with any machine learning classifiers. This is in contrast with the CPOP method, which can only be applicable to a relatively small number of features and is implemented by penalized regression and may not be as easily adaptable with other machine learning classifiers, as the authors themselves concede.

We are grateful for your input, as it has enriched our discussion section and facilitated a more comprehensive understanding of our methodology in the broader context of existing strategies.

**Minor comments:
1. Figure 1c. To me this section is a “Classification” step, rather than an “Integration” step, as it contains model training + integration of results etc.**

**Response:** Thank you for your insightful feedback. We concur with your perspective that this section encapsulates more than mere integration and indeed embodies elements of model training and result consolidation, thus making it more akin to a "Classification" step. Considering this, we have amended the title of this section in Fig 1c from "Integration" to "Classification". The corresponding changes have also been reflected in the main body of the text as well.

**2. It will be good that the authors can include a conclusion paragraph at the end of the manuscript to summarize the key take home messages of the paper.**

**Response:** Thank you for your valuable feedback. We agree with your suggestion, and we appreciate the importance of including a concluding paragraph to underscore the key takeaways from our study. We have added a Conclusions section to the end of our manuscript (page 20, line 874) to succinctly summarize our findings, the implications, and their contribution to enhancing phenotype prediction performance in heterogeneous datasets. This addition, we believe, will enhance the clarity and impact of our research, providing readers with a clear and concise summary of our key messages.

**3. It will be good to group the different methods in Figure 3-5 like Figure 6.**

**Response:** Thank you for pointing this out. Accordingly, we have revised Fig 3-5 to align with this grouping style, which we believe will enhance the understandability and comparability of our results.

**Response to reviewer #2**

Thank you very much for your thoughtful and constructive comments! They help to improve the manuscript significantly. In the revision, we followed all your suggestions and addressed all the points with details shown below (your comments are in bold).

**1. The authors indicated that the comBat normalization improved the prediction performance of machine learning classifier when heterogeneous populations presented, and could successfully remove batch effects within the same population. When “the methods” + “comBat”, the prediction performances were improved. But both “the methods” and “comBat” are existing methods, what is the biggest innovation of this study?**

**Response:** We appreciate your time and effort in reviewing our manuscript and for the pertinent question concerning the innovative aspect of our study.

Our research indeed leverages existing methods – different integration methods and ComBat normalization - but the novelty arises from the comprehensive workflow we've developed to address a challenging issue in the field of genomic studies: heterogeneity. We have investigated different effective strategies to integrate omics data under a wide range of heterogeneity. Our work has provided insights into how different types of heterogeneities affect machine learning classifier prediction accuracy and how various merging and integration methods combined with normalization can excel under specific conditions.

One of the innovative highlights is our application of combining normalization with integration methods, which markedly improves prediction performance in the face of heterogeneous populations and successfully mitigates batch effects. We have demonstrated the effectiveness of this approach through realistic applications on colorectal cancer and tuberculosis studies.

Furthermore, our exploration of rank aggregation methods for boosting phenotype prediction performance adds an innovative dimension to our study. This approach aligns with the current trend of utilizing ensemble learning, and our findings suggest that it has comparable performance to other ensemble learning methods.

In conclusion, our study's innovation resides not only in the new workflow developed but also in the novel amalgamation and application of existing tools to yield enhanced performance in cross-study phenotype predictions. We hope this clarifies your concern, and we also added a paragraph to the end of the introduction section (page 3, line 70) to address the innovation of our study to make it clearer. We welcome further questions to help improve our work.

**2. I tried to understand the method details, but failed. Please provide more details of the methods, especially for the newly developed part. Otherwise, it is hard to judge whether the new approach is worthwhile.**

**Response:** Thank you for your insightful feedback. We appreciate your suggestions which have led us to reconsider how we can make our methodology more accessible and straightforward. We are truly sorry for any difficulties you encountered while navigating through our methods in the original manuscript.

In response to your comments, we have comprehensively revised the Methods section to improve clarity, detail, and structure. Here is a summary of the adjustments we have made:

1. We begin the section with a broad overview of the workflow consists of three stages used in our simulation studies, succinctly explaining the entire pipeline. (page 3, line 83)
2. We then delve into the specifics of each stage within the workflow. The first stage - the Simulation Stage, is described with explicit details for the three distinct simulation scenarios. (page 5, line 171)
3. The next part elucidates the settings for the Naïve and Combat Normalization, discussing whether the training data has been normalized. (page 8, line 284)
4. Subsequently, we outline the third stage - the Classification Stage (page 8, line 312). Here, we have expanded on the machine learning classifiers used in our study, with main text results from random forests classifiers and supplementary material showcasing results from other classifiers. We also discuss the ensemble weighted learning methods and rank aggregation methods applied during the classification stages. We also added equations and more details in the ensemble weighted learning methods (page 9, line 336) and rank aggregation methods (page 11, line 426) subsections to help readers understand the methods thoroughly.
5. Beyond the simulation studies, we explain our methods for real data applications on the Colorectal Cancer (CRC) metagenomics data (page 12, line 466) and Tuberculosis (TB) gene expression data (page 12, line 491). In these sections, we detail the information about the datasets, data processing, and the integration methods applied for making predictions.

We have substantially rewritten most paragraphs and offered additional information to ensure clear understanding. It's worth noting that these changes are not merely cosmetic; they serve to improve the comprehensibility and transparency of our approach.

We think these revisions make the Methods section more informative and provide you with a clearer understanding of our research. We are confident that the improvements we have made will assist you in evaluating the novelty and validity of our work.

We kindly ask for your consideration in accepting our manuscript, and we are open to any further comments you may have.
